# Corticolimbic Circuitry in Chronic Pain Tracks Pain Intensity Relief Following Exposure In Vivo

**DOI:** 10.1016/j.bpsgos.2021.03.004

**Published:** 2021-04-08

**Authors:** Inge Timmers, Vincent G. van de Ven, Johan W.S. Vlaeyen, Rob J.E.M. Smeets, Jeanine A. Verbunt, Jeroen R. de Jong, Amanda L. Kaas

**Affiliations:** a Department of Rehabilitation Medicine, Maastricht University, Maastricht, the Netherlands; b Department of Cognitive Neuroscience, Maastricht University, Maastricht, the Netherlands; c Experimental Health Psychology, Maastricht University, Maastricht, the Netherlands; d CIR Revalidatie, Zwolle, the Netherlands; e Adelante Centre of Expertise in Rehabilitation and Audiology, Hoensbroek, Limburg; f Intergrin, Geleen, the Netherlands; g Department of Anesthesiology, Perioperative, and Pain Medicine, Stanford University, Palo Alto, California; h Department of Health Psychology, Leuven, Belgium

**Keywords:** Chronic pain, Corticolimbic circuitry, Exposure in vivo, Pain intensity, Resting-state fMRI

## Abstract

**Background:**

A subset of patients with chronic pain who receive exposure in vivo (EXP) treatment experience clinically relevant relief of pain intensity. Although pain relief is not an explicit therapeutic target, it is important to understand how and why this concomitant effect occurs in some patients but not others. This longitudinal study therefore aimed to characterize brain plasticity as well as to explore pretreatment factors related to pain relief.

**Methods:**

Resting-state functional magnetic resonance imaging data were acquired in 30 patients with chronic pain. Twenty-three patients completed EXP, and 6-month follow-up data were available in 20 patients (magnetic resonance imaging data in 17 patients). Pain-free control data were acquired at two time points (*n* = 29, *n* = 21). Seed-based resting-state functional connectivity (rsFC) analyses were performed, with seeds in the amygdala, hippocampus, and nucleus accumbens.

**Results:**

Pain relief after EXP was highly variable, with 60% of patients reporting a clinically relevant improvement. Amygdala rsFC with the middle frontal gyrus decreased significantly over time in patients but was not associated with pain relief. In contrast, greater pain relief was associated with greater decreases over time in hippocampus rsFC with the precuneus, which was related to reductions in catastrophizing (EXP therapeutic target) as well. Greater pain relief was also associated with lower pretreatment rsFC between nucleus accumbens and postcentral gyrus.

**Conclusions:**

While changes in hippocampus rsFC were associated with pain relief after EXP, pretreatment nucleus accumbens rsFC showed potential prognostic value. Our findings further support the importance of corticolimbic circuitry in chronic pain, emphasizing its relation to pain relief and identifying potential underlying mechanisms and prognostic factors, warranting further testing in independent samples.

Chronic pain is a significant health problem and a leading cause of disability. For a subset of patients, pain-related distress (e.g., worries, fears) is elevated, contributing to the development and maintenance of chronic pain (1,2). Accordingly, chronic pain is nowadays characterized as an emotional state (3,4) with potential alterations in aversive behavioral learning (5) and in reward/motivational circuits that may further strengthen affective pain mechanisms (6,7). The cognitive behavioral treatment exposure in vivo (EXP) has been successful in reducing fears and disabilities in several patients with pain, including chronic low back pain (cLBP) and complex regional pain syndrome type I (CRPS-I) (10, 11, 12, 13, 14, 15, 8, 9).

As a clinical analog of extinction learning, EXP exposes patients to fear-provoking daily-life movements and activities while examining and challenging worries, interpretations, and expectations about movement and pain as a signal for (re)injury to improve daily-life functioning (16,17). Importantly, pain intensity is not a therapeutic target in EXP. Its aim is rather to move the patient’s focus away from prioritizing immediate pain control to pursuing valued life goals (1,18). Nonetheless, roughly half of the patients do report clinically relevant pain relief after EXP (10,11). Understanding how and why pain relief occurs in some but not others would help understand and optimize treatment approaches to maximize pain relief, even when not targeted.

Some pain reduction after EXP may be expected based on conceptual models (1) and meta-analyses showing small to medium correlations between pain-related fear and pain intensity (19). However, pain intensity does not appear to moderate the relationship between pain-related fear and disability (20), and it remains unclear whether and how reductions in EXP therapeutic targets (i.e., fears, worries) are related to reductions in pain intensity. However, there is a wealth of literature showing that pain experiences are subject to modulation by cognitive and emotional factors (e.g., fears, appraisals, motivation), suggesting that targeting fears and worries may affect pain intensity through such modulations. Interactions within corticolimbic circuitry, reciprocally connected to the brainstem, underlie these modulations (21, 22, 23, 24), with the limbic nuclei amygdala, hippocampus, and nucleus accumbens (NAc) as key players. Whether pain relief after exposure-based treatments is also related to engagement of such circuitry has not been addressed yet and will be a primary focus of this study.

Few studies have investigated the neural correlates of exposure-based treatments. We recently observed that after EXP, individuals with cLBP showed altered neural responses to pain-related fear-evoking visual stimuli in sensorimotor and cognitive/affective regions (e.g., ventro/dorsolateral prefrontal cortex [PFC], posterior cingulate cortex [PCC]) (25). Another study in posttraumatic stress disorder reported enhanced resting-state functional connectivity (rsFC) after exposure-based treatment between the amygdala and orbitofrontal cortex and between the hippocampus and medial PFC in patients compared with control participants (26). Other studies have investigated brain changes in patients with chronic pain following cognitive behavioral treatment (CBT) (including elements of EXP) and have further implicated the PFC and subcortical limbic structures (e.g., amygdala) as important mediators of treatment responses (27, 28, 29). In addition, pretreatment PCC rsFC within the dorsal attention network was found to predict treatment changes in anxiety and pain intensity after CBT (30). After nonpsychological treatments, circuitry including the default mode network (DMN) and sensorimotor network have been implicated in pain relief (31, 32, 33), and rsFC with these networks may have prognostic value, too (32,34). Whether pain relief is associated with similar rsFC pretreatment patterns and changes after exposure-based treatments remains to be tested.

To address these gaps, the current longitudinal study probed limbic rsFC correlates of pain relief in patients with chronic pain after EXP. We included patients with cLBP and CRPS-I, two pain types in which EXP has proven successful in reducing disability (10,14) and for which EXP is standard care in our center. Our focus was specifically on amygdala, hippocampus, and NAc rsFC with the rest of the brain because of their role in affective processing, aversive/threat learning, and reward/motivational processing of pain. Data were collected before and after EXP and 6 months after. A control group without chronic pain (and no treatment) was added for comparison. Our main objectives were to characterize pain relief after EXP in terms of self-reports and rsFC and to explore whether pretreatment rsFC was associated with pain relief. We expected varying degrees of pain intensity relief and expected that approximately half of the patients would experience a clinically relevant reduction in pain [40%–60%, based on (10,11)]. We hypothesized that pain relief would be associated with changes in rsFC over time as well as with pretreatment rsFC. Specifically, we expected that amygdala, hippocampus, and NAc rsFC networks and their connectivity with regions of the DMN or sensorimotor network would demonstrate such associations.

## Methods and Materials

### Overall Study Procedure

This study presents data of a larger study, BrainEXPain. BrainEXPain was approved by the Medical Ethical Committee of Maastricht University Hospital/Maastricht University (MUMC+/UM) and registered at ClinicalTrials.gov (NCT02347579).^1^ Part of this dataset was published before (i.e., involving the cLBP sample only) (25), but the resting-state functional magnetic resonance imaging (rs-fMRI) data have not been described elsewhere.

Patients were recruited via the Department of Rehabilitation Medicine of MUMC+/Adelante Rehabilitation Center, where patients were seen for consultation with a physiatrist (confirming pain diagnosis and excluding alternative diagnoses). Informed consent was obtained at study enrollment. Before scanning, participants filled in questionnaires online (Qualtrics, Provo, UT; Qualtrics.com). The first study visit was scheduled before any treatment (i.e., pre-EXP). Then, patients underwent a multidisciplinary pain screening and pain education and started the exposure sessions (if eligible for treatment). At the end of the treatment, a post-treatment study visit was scheduled as well as a follow-up study visit 6 months after the end of the treatment (post-EXP and FU-EXP, respectively). For pain-free controls, two study visits were scheduled with a time interval matching the patients’ pre- to post-EXP interval (Table 1 and Table S2). Participants received €15 per visit and travel reimbursement for their participation.

**Table 1:** Demographics of the Sample That Was Scanned/Analyzed at Each Time Point

Demographic	Time Point	Patients With Chronic Pain, Mean (SD) or *n*	Pain-Free Volunteers, Mean (SD) or *n*	Group Equivalence Testing
Sample Size	Pre-EXP	30	29	N/A
	Post-EXP	18	21	
	FU-EXP	17	N/A	
Age, Years	Pre-EXP	40.6 (12.7)	42.0 (12.6)	*F*_1,57_ = 0.18, *p* = .67
	Post-EXP	37.3 (12.1)	39.7 (12.0)	*F*_1,37_ = 0.38, *p* = .54
	FU-EXP	38.6 (11.9)	N/A	N/A
Gender	Pre-EXP	19 male, 11 female	15 male, 14 female	χ^2^_1__,_*_n_*_= 59_ = 0.81, *p* = .37
	Post-EXP	12 male, 6 female	11 male, 10 female	χ^2^_1,_*_n_*_= 39_ = 0.82, *p* = .37
	FU-EXP	12 male, 5 female	N/A	N/A
Pain Type	Pre-EXP	cLBP: 18; CRPS-I: 12	N/A	N/A
	Post-EXP	cLBP: 10; CRPS-I: 8		
	FU-EXP	cLBP: 10; CRPS-I: 7		
Pain Duration, Months	Pre-EXP	60.4 (92.2)	N/A	N/A
	Post-EXP	76.2 (115.2)		
	FU-EXP	81.2 (117.6)		
EXP Duration, Days	Pre-EXP	54.1 (22.0)	N/A	N/A
	Post-EXP	52.9 (23.3)		
	FU-EXP	53.7 (24.5)		
Medication Use	Pre-EXP	No medication: 21 Paracetamol/NSAID: 5 Opioids: 5 Other^a^: 7	No medication: 27 Paracetamol/NSAID: 1 Opioids: 0 Other: 1	N/A
	Post-EXP^b^	No medication: 15 Paracetamol/NSAID: 0 Opioids: 0 Other: 3	No medication: 21	N/A
	FU-EXP^c^	No medication: 14 Paracetamol/NSAID: 2 Opioids: 1 Other: 2	N/A	N/A

cLBP, chronic low back pain; CRPS-I, complex regional pain syndrome type I; EXP, exposure in vivo; FU, follow-up; N/A, not applicable; NSAID, nonsteroidal anti-inflammatory drug.

a Other medication includes anticonvulsants, anxiolytics, and antidepressants.

b Fourteen patients did not change medication from pre- to post-EXP, and 4 patients had a decrease in medication from pre- to post-EXP.

c Thirteen patients did not change medication from pre- to FU-EXP, 3 patients had a decrease, and 1 patient had an increase in medication from pre- to FU-EXP.

### Participants

Figure S1 presents the recruitment process and all numbers. The Supplemental Methods presents inclusion and exclusion criteria and more details. In brief, the sample consisted of 30 patients referred for EXP after physiatrist consultation (18 with cLBP, 12 with CRPS-I) (Table 1 and Table S1), of which 23 underwent treatment. Post-EXP MRI data were available for 19 patients (56% cLBP, 44% CRPS-I), and FU-EXP MRI data for 17 patients (59% cLBP, 41% CRPS-I). Follow-up self-reported data on pain-related outcomes were available for 20 patients (55% cLBP, 45% CRPS-I) (Figure S1).

The patient group was compared with a sample of 31 pain-free volunteers, matched for age, gender, and handedness. Two control participants were excluded owing to extensive motion during scanning (see MRI Acquisition and Analysis); thus, the final sample consisted of 29 control participants (Table 1 and Figure S1). To match the patient group, 21 control participants underwent a second study visit (Table S2).

### Exposure In Vivo

EXP is standard care for patients with chronic pain presenting with elevated pain-related fear at MUMC+/Adelante. EXP is a form of CBT in which patients are exposed to feared movements and activities, while interpretations and expectations about movement and pain as a signal for (re)injury are examined and challenged. A detailed description of the EXP protocol can be found in Vlaeyen *et al.* (16,17) and Verbunt and Smeets (35).

### Self-reported Outcomes

At all time points, we assessed the following: pain intensity using a 0–10 visual analog scale anchored with “no pain at all” and “worst pain imaginable”; pain-related disability using the Pain Disability Index (36); pain-related fear using an adapted version of the Photograph Series of Daily Activities for Low Back (37), Upper Extremities (38), or Lower Extremities (39); and pain catastrophizing using the Pain Catastrophizing Scale (40).^2^ At follow-up, patients were also asked to rate how successful they found their treatment overall and in terms of decreasing disability, increasing participation, decreasing pain-related distress, and decreasing pain on a visual analog scale ranging from 0 (not successful at all) to 10 (very successful).

### Behavioral Data Analysis

Questionnaire data were analyzed using SPSS (version 24; IBM Corp., Armonk, NY). A general linear model with group (patients-cLBP, patients-CRPS-I, control participants) as between-subjects factor was used to examine pretreatment group differences (pre-EXP) as well as at post-EXP. A repeated-measures analysis with time (pre-EXP, post-EXP, FU-EXP) as a within-subjects factor was used to investigate changes over time in patients. Changes over time were evaluated for clinical relevance. A clinically relevant reduction in pain intensity was defined as a reduction of at least 30% and 2 absolute points compared with pretreatment. These criteria, separately, have been utilized before (34,41), and research supports that a reduction of 30% reflects a clinically important difference as self-reported by patients, irrespective of pretreatment pain intensity (42).

### MRI Acquisition and Analysis

MRI data were collected using a 3T whole-body MRI scanner (Philips Gyroscan Achieva; Philips Healthcare, TX) using a 32-channel head coil. The Supplemental Methods describes acquisition parameters and data analysis details. In brief, after standard preprocessing in CONN (43), we performed denoising procedures, including noise regressors (motion parameters and first derivatives, white matter/cerebrospinal fluid noise components) and bandpass filtering (0.005–0.1 Hz). Datasets with absolute motion exceeding 3 mm/degrees in reference to the first volume were excluded, resulting in three exclusions. First-level analysis then estimated bivariate correlation coefficients between the defined seeds (bilateral amygdala, hippocampus, and NAc, based on Harvard-Oxford atlas regions, thresholded at .25) and their targets (i.e., all voxels in the whole brain). A seed-to-voxel functional connectivity analysis was performed in CONN having group (patients, control participants) as between-subject factor and time (pre-EXP, post-EXP, FU-EXP) as within-subject factor. Main analyses focused on patients’ 1) effects of time (pre- to FU-EXP), 2) correlations between changes in rsFC and absolute changes in pain intensity (as we expected high variability in pain relief), and 3) correlations between pre-EXP rsFC and absolute changes in pain intensity. Effects of time were investigated in control participants as well, as were interaction effects (limited to pre- to post-EXP). All effects were evaluated using a cluster-defining threshold of *p* < .001 and a subsequent cluster-level threshold of false discovery rate–adjusted *p* (*p-FDR)* < .05. For relevant clusters, correlation coefficients were extracted and transformed to *z* scores using Fisher’s transformation and further explored in SPSS.

## Results

### Changes Over Time in Self-reported Outcomes

**Figure 1:**
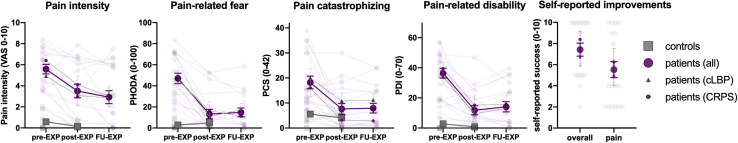
Changes in self-reported outcomes across groups. Presented are mean and standard error in addition to individual data (faded colors) for the control participants (gray) and for the patients (purple); averaged as well as separate for each patient group. cLBP, chronic low back pain; CRPD, complex regional pain syndrome; EXP, exposure in vivo; FU, follow-up; PCS, Pain Catastrophizing Scale; PDI, Pain Disability Index; PHODA, Photographs of Daily Activities; VAS, visual analog scale.

On average, patients showed significant improvements in pain intensity, pain-related fear, pain catastrophizing, and pain-related disabilities (Figure 1). All four domains showed significant effects from pre- to post-EXP and from pre- to FU-EXP and no differences from post- to FU-EXP (Table S3). Because we are mostly interested in persistent changes, the following analyses therefore mainly focus on pre- to FU-EXP changes. In addition, no group differences across pain type (cLBP or CRPS-I) (Table S4) at pre-EXP or interactions between pain type and time (Table S3) were observed, indicating that the patient groups did not differ from each other and in their changes over time. At post-EXP, both patient groups reported higher levels of pain intensity, pain catastrophizing, and pain-related disability than control participants but did not differ anymore in terms of pain-related fear (Table S4). Hence, the two patient groups were taken together in the main rsFC analyses. See Supplemental Results for more details.

Pain relief was highly variable across patients, with an average reduction of −2.0 (range −6.4 to +3.2) from pre- to post-EXP and −2.7 (range −7.6 to +3.0) from pre- to FU-EXP (Figure 1 and Table S3). Moreover, a clinically relevant reduction in pain intensity was observed in 60% of patients (both pre- to post-EXP, and pre- to FU-EXP).

### Changes Over Time in rsFC

#### Group-Level Changes Over Time

**Figure 2:**
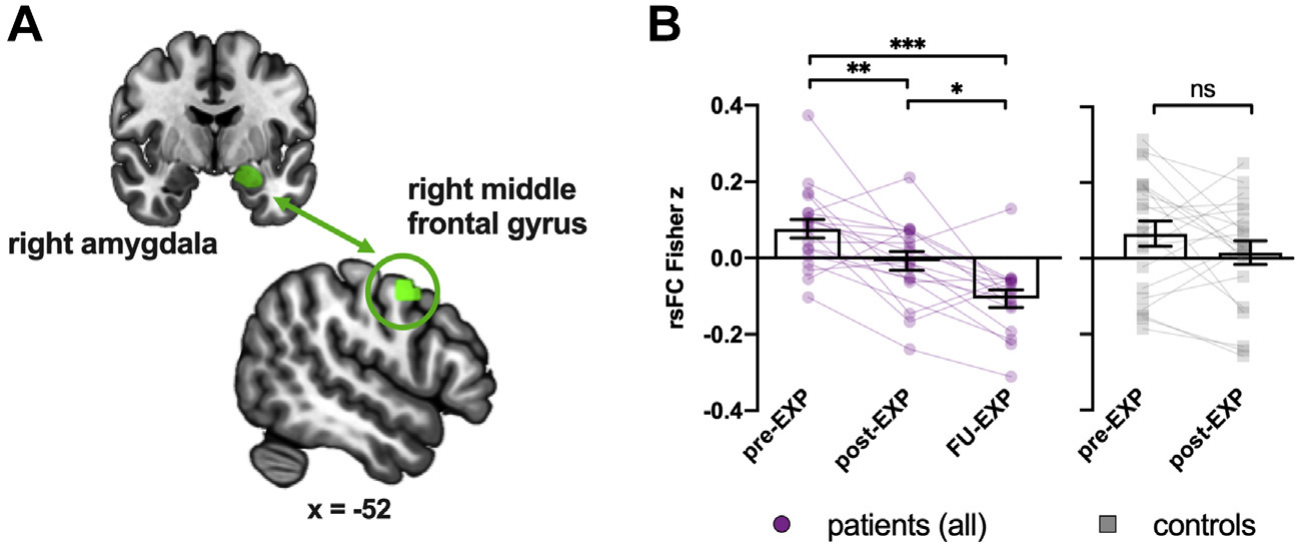
Group-level changes after exposure in vivo (EXP) in resting-state functional connectivity (rsFC). **(A)** The rsFC pair showing a significant difference between pre- and follow-up (FU)-EXP. **(B)** Extracted parameter estimates showing that rsFC between the right amygdala (seed) and the right middle frontal gyrus was decreased after treatment for patients, while no significant change over time was observed for control participants. Individual data are plotted as well using faded colors. ∗*p-corr* < .05, ∗∗*p-corr* < .01, ∗∗∗*p-corr* < .001. ns, not significant.

On average, decreases were observed from pre- to FU-EXP in rsFC between the right amygdala and a cluster in the right precentral/middle frontal gyrus (cluster *p-FDR* = .04; rsFC changed sign from positive to negative from pre- to FU-EXP) (Figure 2 and Table S5). The decrease in rsFC was described by a linear effect (*F*_1,37_ = 51.51, *p* < .001), and post hoc comparisons showed that there was a significant decrease from pre- to post-EXP (*p-corr* = .008) and post- to FU-EXP (*p-corr* = .03). The change in rsFC did not correlate with changes in pain intensity (*r* = −.20, *p* = .47). In control participants, the rsFC between these pairs did not change significantly from pre- to post-EXP (*F*_1,20_ = 2.30, *p* = .15), but there was no significant interaction effect between group and time (limited to pre- to post-EXP; *F*_1,37_ = 0.79, *p* = .38). No group differences were observed at pre- and post-EXP (pre-EXP: *F*_1,57_ = 0.11, *p* = .74; post-EXP: *F*_1,37_ = 0.26, *p* = .61).

#### rsFC Changes Associated With Changes in Pain Intensity

**Figure 3:**
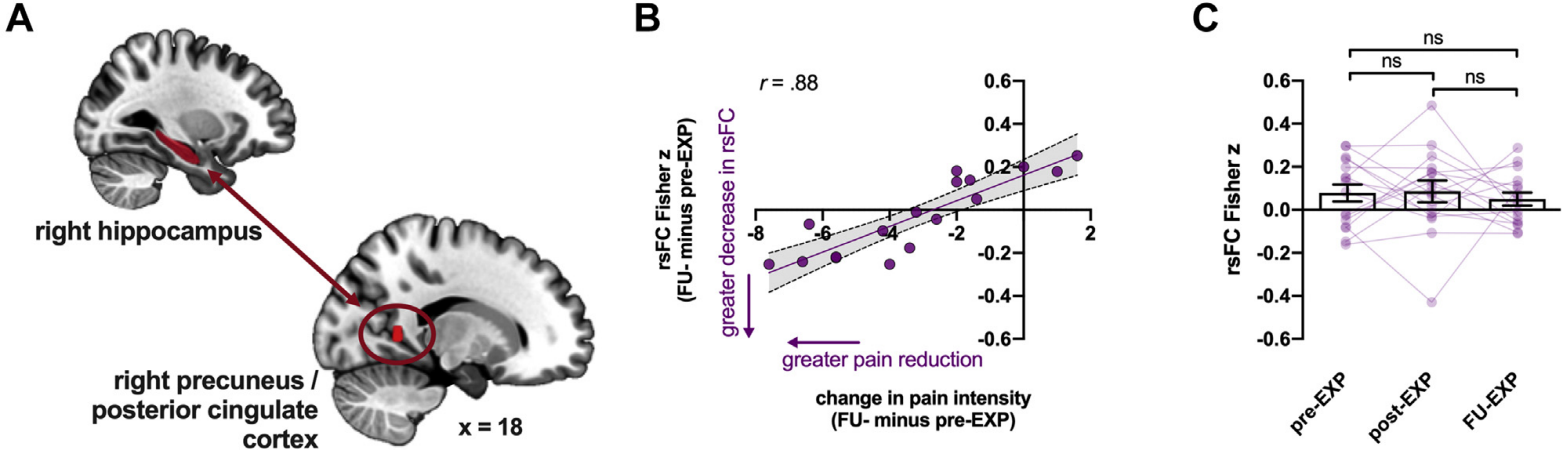
Resting-state functional connectivity (rsFC) change associated with pain relief. **(A)** The rsFC pair in which changes over time were significantly correlated with pain relief: right hippocampus (seed) rsFC with the right precuneus/posterior cingulate cortex. **(B)** The extracted parameter estimates are plotted against changes in pain intensity to visualize the association. **(C)** Extracted parameter estimates across time points showing that on a group level, rsFC did not change significantly. Individual data are plotted as well using faded colors. EXP, exposure in vivo; FU, follow-up; ns, not significant.

To investigate rsFC changes associated with pain relief, we correlated changes in rsFC with changes in pain intensity (FU-EXP − pre-EXP; *n* = 17). Greater reductions in pain intensity were associated with greater decreases in right hippocampus rsFC with precuneus/PCC (cluster *p-FDR* = .04) (Figure 3 and Table S5). This correlation remained significant when expressing pain reduction in percentages rather than in absolute differences (*r* = −.83, *p* < .001). On a group level, rsFC did not significantly change over time (main effect time: *F*_1.8,27.5_ = 0.24, *p* = .77) (Figure 3C).

#### Pain Relief and Therapeutic Targets

To explore whether pain relief was associated with changes in primary therapeutic targets, we performed correlation analyses between pain intensity changes from pre- to FU-EXP with changes in fear, catastrophizing, and disability from pre- to FU-EXP. Taking pre-EXP pain intensity into account, reductions in pain intensity were significantly correlated with reductions in catastrophizing (*r*_*p*_ = .57, *p* = .03), but not significantly with reductions in fear and disability (fear: *r*_*p*_ = .44, *p* = .10; disability: *r*_*p*_ = .40, *p* = .14).

**Figure 4:**
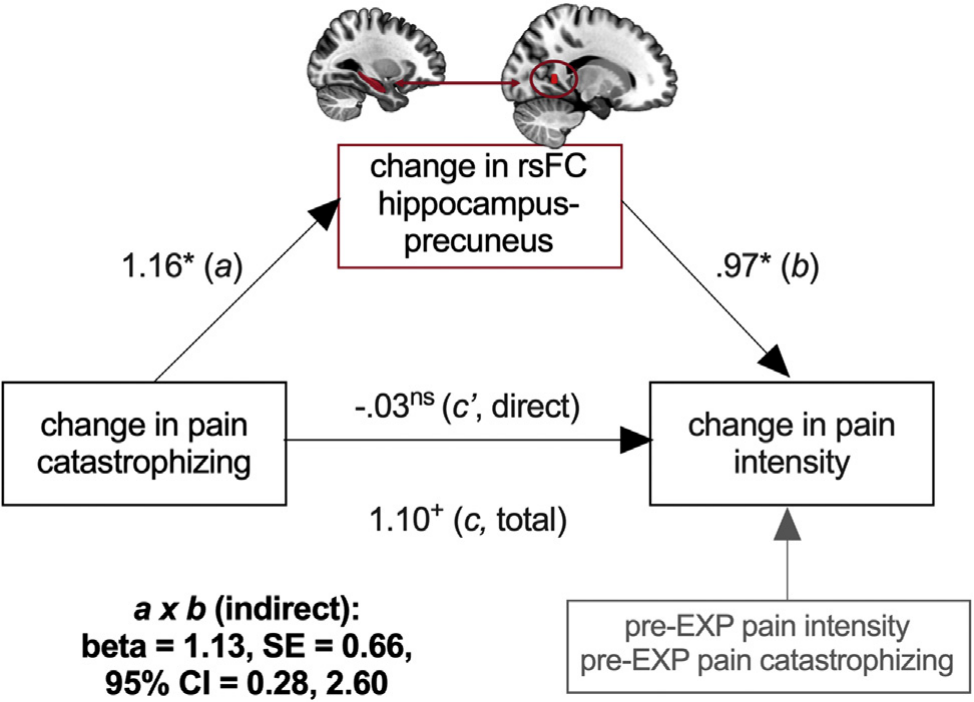
Explorative mechanistic role for hippocampus-precuneus/posterior cingulate cortex (PCC) resting-state functional connectivity (rsFC). Changes in pain catastrophizing are also related to hippocampus-precuneus/PCC rsFC, and the relationship between changes in pain catastrophizing (therapeutic target) and pain relief is explained by changes in rsFC between the hippocampus and precuneus/PCC. Pretreatment pain intensity and pain catastrophizing are added as covariates. Presented are the standardized coefficients of the separate paths as well as the indirect effect (95% confidence interval [CI] from bootstrap analysis). ^+^*p* = .05, ∗*p* < .05, exposure in vivo; ns, not significant.

In addition, follow-up analyses showed that changes in catastrophizing were also correlated with changes in hippocampus-precuneus/PCC rsFC (path *a*) and that the relationship between changes in catastrophizing and in pain was mediated by changes in hippocampus-precuneus rsFC (indirect path *ab*) (Figure 4), even when controlling for pre-EXP pain intensity and catastrophizing.

### Associating Pain Relief With Pretreatment rsFC

To investigate whether pain relief was associated with pretreatment data, we correlated patients’ pre-EXP data with pain intensity changes (FU-EXP − pre-EXP; *n* = 20).

**Figure 5:**
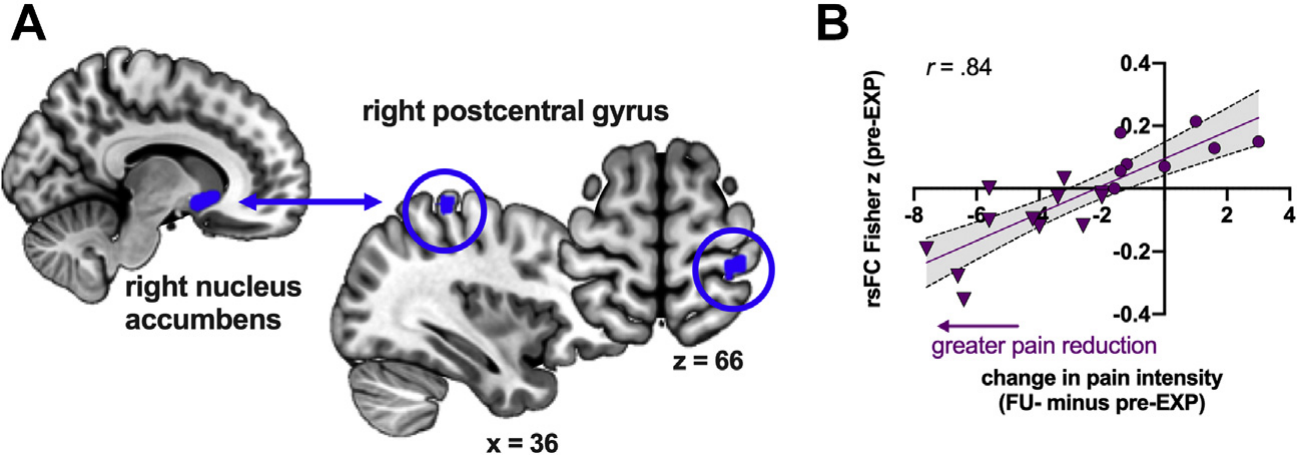
Pretreatment resting-state functional connectivity (rsFC) is associated with pain relief in patients. **(A)** Anatomical locations of the seed in the right nucleus accumbens and the cluster in the right postcentral gyrus. **(B)** The correlation is visualized, showing that greater pain reductions after treatment are associated with lower (more negative) rsFC between the right nucleus accumbens and right postcentral gyrus at pretreatment. Patients experiencing clinically relevant pain relief are presented as a triangle, while those who did not are presented as a circle. EXP, exposure in vivo; FU, follow-up.

Changes in pain intensity were not correlated with any of the self-reported baseline characteristics or pain-related variables (all *ps* > .05) (Supplemental Results). Lower right NAc rsFC with the right postcentral gyrus at pre-EXP was associated with greater pain reductions (cluster *p-FDR* < .001) (Table S5); rsFC changed sign from positive to negative with greater pain reductions (Figure 5). The correlation remained significant when using percentages of pain reduction (*r* = .76, *p* < .001) and when controlling for pre-EXP pain intensity and pain catastrophizing (*r*_*p*_ = .83, *p* < .001). Further examinations showed that there were no pre-EXP differences in this rsFC when comparing all patients and control participants (*n* = 30 vs. *n* = 29; *F*_1,57_ = 0.01, *p* = .93). There were also no signs that this rsFC changed over time in patients (*F*_1.5,23.2_ = 0.96, *p* = .38).

## Discussion

While pain intensity is not an explicit therapeutic target of EXP, pain relief after EXP is nevertheless observed in a subset of patients with chronic pain. To understand how and why pain relief occurs in some but not others, we longitudinally examined rsFC in individuals with chronic pain receiving EXP, focusing on subcortical limbic nuclei involved in affective/motivational processing and learning. Our main findings are 1) at a group level, patients showed decreases in amygdala rsFC with the dorsolateral PFC (dlPFC) from pretreatment to 6-month follow-up, not associated with pain relief; 2) at an individual patient level, greater pain relief after EXP was associated with greater decreases in hippocampus rsFC with the posterior medial cortex (PMC; precuneus/PCC); 3) this hippocampus-PMC rsFC decrease mediated the relationship between pain catastrophizing changes and pain intensity changes (independent of pretreatment levels); and 4) greater pain relief was associated with lower pretreatment NAc rsFC with the postcentral gyrus (primary somatosensory cortex). Thus, while hippocampus rsFC changes were associated with pain relief after EXP, pretreatment NAc rsFC showed associations with pain relief, signaling potential prognostic value.

In line with previous literature (10,11), pain relief following EXP was highly variable, with 60% of patients reporting clinically relevant improvements in pain lasting at least 6 months. Previous studies suggested that individuals with higher pretreatment pain, anxiety, catastrophizing, and/or affective distress may reach smaller treatment gains (44, 45, 46), although findings are inconsistent. We did not find support for this idea. If anything, it seemed that higher catastrophizing at pretreatment was associated with greater pain relief, but this did not reach significance. The fact that our patients were referred to multidisciplinary screening after initial consultation potentially biased our sample toward higher levels of fears or worries than other (e.g., community-based) samples of patients with chronic pain. Nonetheless, 60% experienced pain relief, and pretreatment self-reported data were not associated with pain relief.

Pretreatment resting-state connectivity, however, was associated with subsequent pain relief. In particular, lower pretreatment NAc-sensorimotor rsFC was associated with greater pain relief. Previous studies have proposed an important role for the NAc, and more generally, the reward/motivational system, in chronification—or “stickiness”—of pain (6,7,47, 48, 49, 50, 51), suggesting that patients with altered reward or aversion processing may be at a higher risk for developing chronic pain and more resistant to treatment. Moreover, NAc (part of the ventral striatum) is a key region for analgesia (52,53), including placebo analgesia (i.e., pain relief related to treatment context/expectancies) (54), potentially through its involvement in (reward-related) prediction errors (55,56). In our study, less pain relief was achieved by those individuals with stronger connectivity between the reward/motivational and the sensorimotor network before treatment. Although anatomical connections between the sensorimotor cortex and NAc are likely indirect (57), we hypothesize that the enhanced functional connectivity points to a differential role for sensorimotor input in evaluating prediction errors or to a stronger motivational drive or expected reward for certain somatosensory signals. Furthermore, our findings suggest that pretreatment rsFC could help predict which individuals may be more susceptible to experiencing pain relief. This would have drastic prognostic implications and could aid individually tailored treatment plans (58). Future studies with independent samples are needed to test whether this rsFC profile carries prognostic value. If this is the case, pain relief may be optimized if this enhanced functional connectivity would be addressed before (or early on during) EXP. Future studies could investigate whether analysis of somatosensory processing (e.g., tactile sensitivity, acuity, proprioception) could bring about relevant insights. Moreover, as maximizing the mismatch between the expectancy and the actual experience is thought to optimize inhibitory learning (59), it could be interesting to examine potential links between such expectancy violations (or prediction errors) during EXP and confirmation biases in aversive learning (i.e., tendency for stronger updating of expectancies when experiences are more consistent with initial expectancies). Research has shown that individual differences in such biases are reflected in updating of pain-anticipatory brain activation (i.e., involving sensorimotor regions and dorsal striatum) (60). Whether the NAc-sensorimotor rsFC is reflective of individual differences in confirmation biases, such that those with stronger rsFC have stronger biases and hence achieve less updating of pain-anticipatory circuitry and in turn less pain relief, warrants further testing.

Changes in hippocampus-PMC circuitry after EXP correlated with pain relief. PMC (including precuneus, PCC, and retrosplenial cortex) has been implicated in fear conditioning (61) and extinction (62). It represents a functional core within the DMN, involved in self-referential reflective processing (63) and other self-oriented functions (e.g., autobiographical memory retrieval, reward-outcome monitoring, action-outcome learning), partly through hippocampus connections (64, 65, 66). Indeed, the hippocampus has often been reported to be functionally connected to the DMN (67, 68, 69). In nonpsychological treatment studies, posterior DMN connectivity was related to pain intensity (33,34). Changes in DMN circuitry after a brief CBT intervention in healthy individuals were also related to changes in pain intensity and unpleasantness (70). Our data extend these findings, highlighting the importance of changes in hippocampus-PMC interactions for achieving pain relief after EXP (i.e., the clinical analog of extinction learning). Whether this finding is reflective of broader hippocampus-DMN rsFC remains to be tested.

Further exploring how hippocampus-PMC connectivity may be associated with pain relief, a mediation analysis revealed that reductions in catastrophizing (EXP therapeutic target) were also associated with hippocampus-PMC rsFC and that the relationship between changes in catastrophizing and changes in pain intensity was explained by changes in hippocampus-PMC rsFC. Note, though, that the hippocampus-PMC rsFC mediator was selected based on its correlation with pain relief, which can be considered circular and likely creates a bias. Although warranting further investigations, this analysis provides preliminary support for a potential mechanistic role of hippocampus-PMC circuitry: independent of pretreatment catastrophizing and pain, individuals with greater changes in catastrophizing showed greater pain relief, mediated by changes in hippocampus-PMC coupling. Previous research has shown that changes in catastrophizing are underlain by different substrates, including reduced connectivity between the insula and sensorimotor regions (71), suggesting that different facets of clinical improvements are underlain by differential patterns of brain plasticity.

Finally, on a group level, patients showed a general time effect in amygdala rsFC, which was absent in control participants (although there was no significant interaction when limiting to pre- to post-EXP changes). Amygdala rsFC with the dlPFC extending into the premotor cortex (i.e., middle frontal gyrus) decreased linearly over time. Because this effect was not associated with (changes in) pain intensity, it appears to be a general correlate of EXP. Other studies have also pointed toward the dlPFC as a key region in (chronic) pain (72), associated with modulation and perceived control of pain (73,74) and catastrophizing (75). dlPFC structural and functional changes during rest and during cognitive tasks have been observed after treatment (76,77). The decrease over time in dlPFC rsFC with the amygdala (i.e., a core center for threat processing and learning) (78,79) may reflect reduced engagement (or inhibition) of top-down pain modulatory regions by the amygdala. However, this group-level effect was not associated with pain relief and may even reflect more general effects of time.

Our findings need to be interpreted in light of some considerations. The first is our relatively small sample. Data were acquired within a clinical setting, and not all patients commenced treatment after the multidisciplinary pain screening. Regardless, findings were consistent across different analytical approaches (continuous vs. dichotomous) and robust to different definitions of pain relief (absolute vs. percentage reductions). However, we cannot exclude the possibility that data, in particular the correlations, are overfitted. Second, our sample consisted of two pain types: cLBP and CRPS-I. Although they are quite different in their clinical phenotype, we did not find any difference in their clinical presentation at pretreatment or changes over time, in line with the idea that EXP taps into transdiagnostic mechanisms (i.e., fears, catastrophizing, avoidance) unspecific to pain type. This suggests generalizability of our findings across pain phenotypes, although this needs to be formally examined. Finally, no control treatment was included, precluding any conclusions about a potential causal relation to EXP, as we cannot rule out whether the findings reflect placebo effects or the natural temporal course of chronic pain. Studies including more than one treatment arm or phase could shed more light on this topic.

Taken together, our findings delineate the importance of corticolimbic connectivity in pain relief after EXP. Amygdala and hippocampus circuitry showed signs of plasticity after treatment, with a potential mechanistic role for pain-related memory circuitry (hippocampus-PMC) in achieving pain relief after EXP. In contrast, NAc circuitry at pretreatment showed associations with pain relief, suggesting a catalytic role for reward-sensorimotor coupling in achieving pain relief after EXP. This points to potential prognostic value, warranting further testing in independent samples. These findings add to our knowledge on the involvement of corticolimbic circuitry in pain relief, generating testable hypotheses for the working mechanisms of and prognostic factors for exposure treatments and may be used to further optimize treatment approaches and prognostic markers for pain relief.

## Footnotes

1 Note that only the changes over time were preregistered, and hence, the investigating of pretreatment data in relation to pain relief is more explorative.

2 Note that these self-reported outcomes have been described in our previous publication as well (25), although then limited to the sample with chronic low back pain.
