# Supplementary material for: Corticolimbic Circuitry in Chronic Pain Tracks Pain Intensity Relief Following Exposure In Vivo
Source: Biol Psychiatry Glob Open Sci. 2021 Apr 8;1(1):28–36. doi: 10.1016/j.bpsgos.2021.03.004 (PMC9616294; doi:10.1016/j.bpsgos.2021.03.004)
Supplement: Supplementary Materials [file mmc1.pdf]

Cortico-Limbic Circuitry in Chronic Pain Tracks Pain Intensity Relief Following Exposure In Vivo

Supplemental Information

Supplemental Methods

page 2-6, including Figure S1, Tables S1-S2

Supplemental Results

Page 6-9, including Figure S2, Tables S3-S5

Supplemental Methods

Participants

If patients were motivated for rehabilitation treatment and eligible for the multi-disciplinary pain screening program, they were invited for the study by the physiatrist. Recruitment was open between January 2015 and August 2017. Participants were then contacted by the research team and were screened for in- and exclusion criteria.

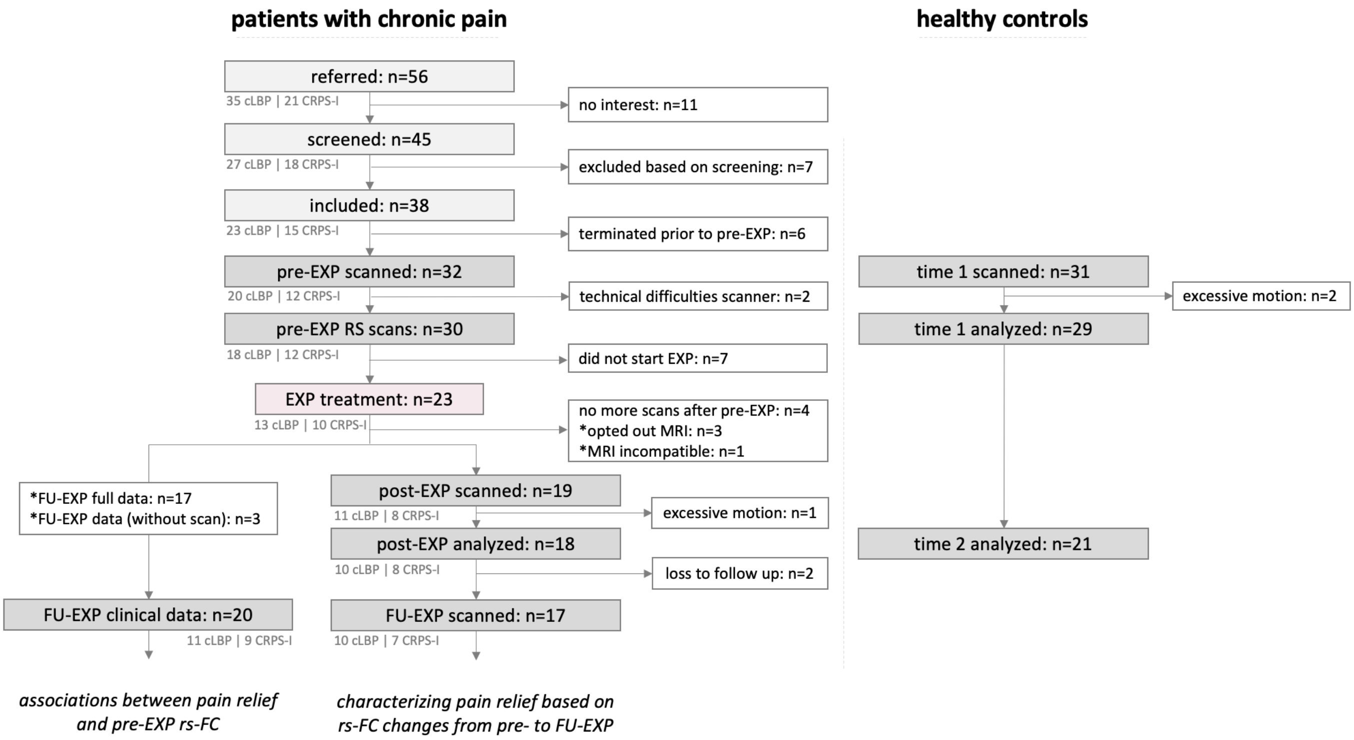

Figure S1: Flowchart of recruitment and assessments for each group. cLBP = chronic low back pain; CRPS-I = complex regional pain syndrome type I; RS = resting-state. EXP = exposure in vivo. FU = follow-up.

Inclusion criteria for patients were age 18-65, stable medication use, experience of non-specific LBP > 6 months or a diagnosis of Complex Regional Pain Syndrome type I (CRPS-I) based on the Budapest criteria (1), and no other diagnosis explaining the symptoms (assessed by the physiatrist). Exclusion criteria were claustrophobia, MRI incompatibility (e.g., pacemaker, pregnancy), and severe psychopathology (Symptom Check List-90; 2). **Table S1** provides more detailed information on the two pain types.

Table S1: Demographics of the patient samples that were scanned/analyzed at each time point

		Patients with cLBP Mean (SD)	Patients with CRPS-I Mean (SD)	Group equivalence testing
**Sample size**	**Pre-EXP**	n = 18	n = 12	n/a
	**Post-EXP**	n = 10	n = 8	
	**FU-EXP**	n = 10	n = 7	
**Age (years)**	**Pre-EXP**	41.8 (11.3)	38.8 (15.0)	$F_{1,28} = 0.39, p = .54$
	**Post-EXP**	39.6 (11.4)	34.4 (13.0)	$F_{1,16} = 0.83, p = .38$
	**FU-EXP**	40.8 (10.7)	35.4 (13.6)	$F_{1,15} = 0.83, p = .38$
**Gender**	**Pre-EXP**	14 m, 4 f	5 m, 7 f	$X^2_{1,n=30} = 4.04, p = .04$
	**Post-EXP**	9 m, 1 f	3 m, 5 f	$X^2_{1,n=18} = 5.51, p = .02$
	**FU-EXP**	10 m, 0 f	3 m, 4 f	$X^2_{1,n=17} = 7.47, p = .006$
**Pain duration (months)**	**Pre-EXP**	52.6 (43.5)	72.1 (138.6)	$F_{1,28} = 0.32, p = .38$
	**Post-EXP**	55.8 (54.0)	101.6 (164.8)	$F_{1,16} = 0.69, p = .42$
	**FU-EXP**	59.4 (53.8)	112.3 (174.9)	$F_{1,15} = 0.82, p = .38$
**Treatment (days)**	**Pre-EXP**	47.6 (16.5)	62.9 (26.3)	$F_{1,19} = 2.69, p = .12$
	**Post-EXP**	45.0 (15.9)	62.8 (28.1)	$F_{1,16} = 2.87, p = .11$
	**FU-EXP**	46.6 (17.9)	63.9 (30.1)	$F_{1,15} = 2.21, p = .16$
**Medication use**	**Pre-EXP**	no medication: n=12<br>paracetamol/NSAID: n=3<br>opioids: n=2<br>other [1]: n=2	no medication: n=7<br>paracetamol/NSAID: n=2<br>opioids: n=3<br>other: n=5	n/a
	**Post-EXP [2]**	no medication: n=9<br>paracetamol/NSAID: n=0<br>opioids: n=0<br>other: n=1	no medication: n=6<br>paracetamol/NSAID: n=0<br>opioids: n=0<br>other: n=2	n/a
	**FU-EXP [3]**	no medication: n=9<br>paracetamol/NSAID: n=1<br>opioids: n=1<br>other: n=1	no medication: n=5<br>paracetamol/NSAID: n=1<br>opioids: n=0<br>other: n=1	n/a

n/a = not applicable; cLBP = chronic low back pain; CRPS-I = complex regional pain syndrome type I; SD = standard deviation; NSAID = non-steroidal anti-inflammatory drugs; [1]other medication includes anticonvulsants, anxiolytics and antidepressants; [2]n=8 patients with cLBP and n=6 patients with CRPS-I did not change medication from pre- to post-EXP, n=2 patients with cLBP and n=2 patients with CRPS-I had a decrease in medication from pre- to post-EXP; [3]n=7 patients with cLBP and n=6 patients with CRPS-I did not change medication from pre- to FU-EXP, n=1 patient with cLBP and n=1 patient with CRPS-I had a decrease, n=1 patient with cLBP had an increase in medication from pre- to FU-EXP.

Controls were recruited through advertisements. Additional exclusion criteria for controls were a history of a chronic pain syndrome, and seeking treatment for a pain condition

in the last six months. Controls were each matched to a patient by age, gender and handedness when possible (to achieve cohort-level matching). Testing and scanning were interleaved with that of patients to avoid confounding due to changes over time (**Table S2**).

Table S2: Time of testing per group, per time point, demonstrating that testing was interleaved

		Mean	Range
**Patients with chronic pain**	**Pre-EXP**	March 2016	February 2015 – July 2017
	**Post-EXP**	August 2016	June 2015 – December 2017
	**FU-EXP**		November 2015 – May 2018
**Pain-free volunteers**	**Time 1**	July 2016	March 2015 – August 2017
	**Time 2**	October 2016	July 2015 – January 2018

We inspected whether the patients who did not start EXP or dropped out of the study (n=10) differed from the patients for whom we did collect self-reported data at six-month follow-up (n=20) in demographics or in pre-EXP pain-related outcomes. They did not differ in age ($F_{1,28}$ = 1.48, p = .23), gender ($X^2_{1,n=30}$ = 0.07, p = .79), pain duration ($F_{1,28}$ = 1.05, p = .32), pain type ($X^2_{1,n=30}$ = 0.63, p = .43), pre-EXP pain intensity ($F_{1,28}$ = 0.13, p = .72), pain-related fear ($F_{1,28}$ = 1.68, p = .21), catastrophizing ($F_{1,28}$ = 2.14, p = .15) or pain-related disabilities ($F_{1,28}$ = 1.61, p = .22).

MRI data acquisition

For the resting-state functional images, a T2*-weighted standard echo-planar imaging (EPI) sequence was used to acquire 40 axial slices (3 mm isotropic) covering the entire cerebral volume, using the following parameters: repetition time/TR=2000ms, echo time/TE=25ms, flip angle/FA=75°, matrix size=120x240, SENSE factor=2. In total, 180 functional volumes were collected, of which the first four volumes were dummy volumes that were discarded from subsequent analysis to avoid T1 saturation effects. Participants were instructed to keep their eyes open and were presented with a grey screen with a fixation point.

T1-weighted anatomical images were acquired using a 3D turbo field echo (TFE) sequence with the following parameters: 170 slices, 1 mm isotropic, TR=8.1ms, TE=3.7ms, FA=8°, matrix size=240x240. The full MR protocol also included task-based functional and diffusion-weighted MRI, and had a duration of approximately 90 minutes.

MRI data analysis

CONN was used as a main data analysis tool (3), which is an SPM-based toolbox (4) (ran in Matlab R2017a).

***MRI data– seed selection*.** The *a priori* selected seeds were the left and right analogues of the amygdala, hippocampus and NAc. Regions of interest (ROIs) were defined based on the Harvard-Oxford subcortical atlas (thresholded at probability of .25), and time courses were averaged across these six atlas regions.

MRI data– preprocessing. Pre-processing included 3D head motion correction, slice scan time correction, segmentation into white matter (WM), grey matter (GM) and cerebral spinal fluid (CSF), normalization to MNI space and spatial smoothing (6 mm using a full-width at half-maximum Gaussian kernel [FWHM]).

MRI data– denoising and first-level analysis. Denoising procedures were performed in CONN and included regression of motion parameters and their first derivatives (12 parameters in total) as well as WM/CSF noise components (estimated using anatomical component-based noise correction, aCompCor (5); 10 parameters in total), linear and quadratic trend removal, and simultaneous band pass filtering (.005-.1Hz). Quality assurance steps were performed to identify outliers: datasets with absolute motion exceeding 3 mm/degrees in reference to the first volume were excluded from analysis (i.e., data from 3 participants were excluded; 1 patient with cLBP, 2 controls). First-level analysis then estimated bivariate correlation coefficients between the defined seeds (time courses were averaged across all voxels in a seed) and their targets (i.e., all voxels in the whole brain).

MRI data– functional connectivity analysis. A seed-to-voxel functional connectivity analysis was performed in CONN having Group (patients, controls) as between-subject (BS) factor and Time (pre-EXP, post-EXP, [FU-EXP]) as within-subject (WS) factor. Main analyses focused on 1) effects of Time in the patient group (pre- to FU-EXP); 2) correlations between changes in rsFC and absolute changes in pain intensity in patients; 3) correlations between rsFC at pre-EXP and absolute changes in pain intensity in patients. Effects of Time were investigated in controls as well, as were interaction effects (limited to pre- to post-EXP). All effects were evaluated using a cluster defining threshold (CDT) of $p < .001$ and a subsequent cluster-level threshold of $p\text{-}FDR < .05$. In case of significant effects, correlation coefficients were extracted and transformed to z-scores using Fisher's transformation. In SPSS, effects were further explored, including via the Hayes mediation macro for SPSS (PROCESS, version 3.0), which

estimates indirect (mediation) effects using bias-corrected bootstrapped confidence intervals (with *N*=5,000 bootstrap resamples, estimating a 95% confidence interval) (6).

Supplemental Results

Changes over time in self-reported outcomes

The change in pain intensity was the most modest (see also **Figure 1**), with an average reduction of 2.0 points on a 0-10 scale (SE 0.48) from pre- to post-EXP, and 2.7 points (SE 0.64) from pre- to FU-EXP. In percentages, this corresponds to on average 34.9% (SE 9.8%) reduction from pre- to post-EXP, and 43.7% (SE 11.4%) reduction from pre- to FU-EXP. In contrast, for pain-related fear the average percentage reduction was 79.9% (SE 5.6%) and 72.6% (SE 7.8%) for pre- to post-EXP and pre- to FU-EXP respectively; for pain catastrophizing 48.4% (SE 12.2%) and 40.1% (SE 16.0%); and for pain-related disability 71.5% (SE 6.1%) and 63.0% (SE 9.0%). Also in self-reported measures, improvements in pain were the most modest with an average score of 5.7 (SE 0.77) on a 0-10 scale. In contrast, overall self-reported improvements were scored with an average of 7.3 (SE 0.63) on a 0-10 scale.

Table S3. Changes over time in the patients

	Pre-EXP Mean (SE)	Post-EXP Mean (SE)	FU-EXP Mean (SE)	Main effect of EXP	Interaction effect Pain Type x EXP	Post-hoc comparisons
**Pain intensity (VAS)**	5.6 (0.5)	3.6 (0.7)	3.3 (0.7)	$F_{1.7,24.3} = 8.11$, $p = .003$	$F_{1.7,24.3} = 0.18$, $p = .81$	Pre > post: $p = .02$<br>Pre > FU: $p = .02$<br>Post > FU: $p > .99$
**Pain-related fear (PHODA)**	50.1 (5.8)	15.6 (5.1)	13.9 (4.7)	$F_{1.4,18.2} = 69.3$, $p < .001$	$F_{1.4,18.2} = 2.09$, $p = .16$	Pre > post: $p < .001$<br>Pre > FU: $p < .001$<br>Post > FU: $p > .99$
**Pain catastrophizing (PCS)**	18.4 (7.2)	7.2 (2.2)	7.1 (2.0)	$F_{1.1,15.1} = 9.23$, $p = .007$	$F_{1.1,15.1} = 0.13$, $p = .75$	Pre > post: $p = .03$<br>Pre > FU: $p = .02$<br>Post > FU: $p > .99$
**Pain-related disabilities (PDI)**	37.2 (3.5)	13.9 (3.2)	12.7 (3.7)	$F_{1.6,22.7} = 34.21$, $p < .001$	$F_{1.6,22.7} = 0.33$, $p = .68$	Pre > post: $p < .001$<br>Pre > FU: $p < .001$<br>Post > FU: $p > .99$
**Self-reported improvement in pain (VAS)**	n/a	n/a	5.72 (0.77)	n/a	$F_{1,16} = 1.42$, $p = .25$*	n/a

Note that post-hoc comparisons are corrected for multiple comparisons using Bonferroni correction. * comparing the two pain types using one-way ANOVA.

Table S4. Differences across patients and controls at pre- and post-EXP

	Controls<br>Mean (SE)	Patients with cLBP<br>Mean (SE)	Patients with CRPS<br>Mean (SE)	Main effect of Group	Post-hoc comparisons<br>(Bonferroni corrected)
Pre-EXP					
**Pain intensity (VAS)**	0.6 (0.1)	5.5 (0.6)	6.0 (0.8)	$F_{2,56} = 75.46$, $p < .001$	cLBP > controls: $p < .001$<br>CRPS-I > controls: $p < .001$<br>cLBP > CRPS-I: $p > .99$
**Pain-related fear (PHODA)**	3.3 (0.9)	54.4 (5.5)	46.2 (7.9)	$F_{2,56} = 52.29$, $p < .001$	cLBP > controls: $p < .001$<br>CRPS-I > controls: $p < .001$<br>cLBP > CRPS-I: $p = .70$
**Pain catastrophizing (PCS)**	6.1 (1.1)	23.1 (3.3)	16.9 (3.1)	$F_{2,56} = 16.65$, $p < .001$	cLBP > controls: $p < .001$<br>CRPS-I > controls: $p < .001$<br>cLBP > CRPS-I: $p > .32$
**Pain-related disabilities (PDI)**	2.2 (1.0)	37.3 (3.5)	40.7 (3.4)	$F_{2,55} = 87.01$, $p < .001$	cLBP > controls: $p < .001$<br>CRPS-I > controls: $p < .001$<br>cLBP > CRPS-I: $p > .99$
Post-EXP					
**Pain intensity (VAS)**	0.1 (0.1)	2.0 (0.9)	4.1 (0.8)	$F_{2,34} = 15.97$, $p < .001$	cLBP > controls: $p = .001$<br>CRPS-I > controls: $p < .001$<br>cLBP > CRPS-I: $p = .61$
**Pain-related fear (PHODA)**	4.9 (1.2)	9.9 (4.6)	16.6 (8.6)	$F_{2,22} = 0.96$, $p = .40$	cLBP > controls: $p > .99$<br>CRPS-I > controls: $p = .55$<br>cLBP > CRPS-I: $p > .99$
**Pain catastrophizing (PCS)**	4.1 (1.1)	10.1 (3.0)	3.1 (1.4)	$F_{2,34} = 3.62$, $p = .04$	cLBP > controls: $p = .06$<br>CRPS-I > controls: $p > .99$<br>cLBP > CRPS-I: $p = .10$
**Pain-related disabilities (PDI)**	0.8 (0.3)	9.2 (2.0)	15.1 (6.8)	$F_{2,34} = 8.54$, $p = .001$	cLBP > controls: $p = .04$<br>CRPS-I > controls: $p = .001$<br>cLBP > CRPS-I: $p = .46$

Changes over time in resting-state functional connectivity

Table S5. Details the rsFC pairs identified across all analyses

	Description of effect	MNI x	y	z	cluster size k	cluster *p-uncorr*	cluster *p*-FDR
Group-level changes over time (minimum $t_{16} = 3.69$, $k = 104$***)***							
right amygdala (seed) with right precentral / middle frontal gyrus	pre-EXP > FU-EXP	-52	2	48	104	.002	.04
rsFC changes associated with changes in pain intensity (minimum $t_{15} = 3.73$, $k = 103$***)***							
right hippocampus (seed) with precuneus / posterior cingulate cortex	positive relation ($r = .88$)	18	-52	2	103	.003	.04
Associations between pre-treatment rsFC and reductions in pain intensity (minimum $t_{18} = 3.61$, $k = 238$***)***							
right nucleus accumbens (seed) with right postcentral gyrus	positive relation ($r = .84$)	36	-36	66	238	.0007	< .001

Pain types. When taking the cLBP and CRPS-I group separately, both groups showed a strong correlation between pain relief and change in rsFC between hippocampus and precuneus/PCC (patients with cLBP: $r = .91$; patients with CRPS-I: $r = .78$) (**Figure S2A**)

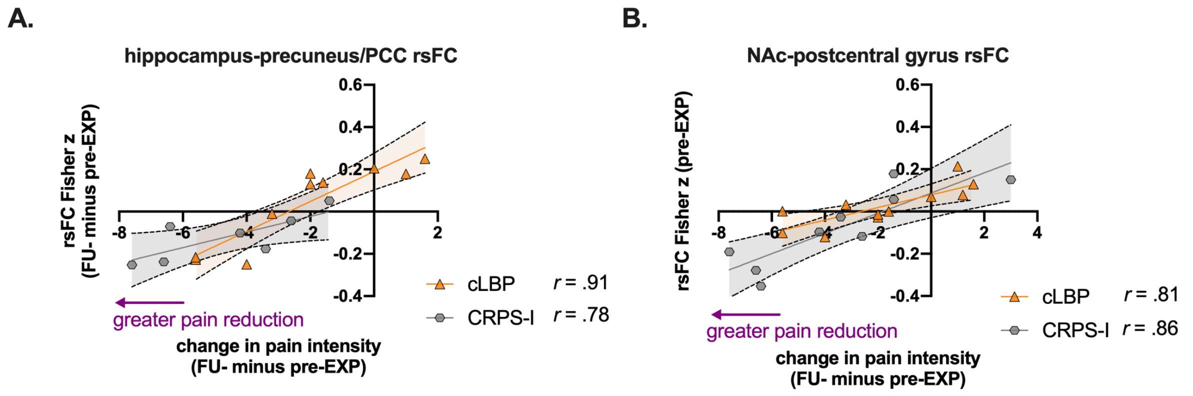

Figure S2. Resting-state functional connectivity change associated with pain relief, per pain type. Per pain type, the extracted parameter estimates, and corresponding fitted line are plotted against changes in pain intensity to visualize the associations separately for each group (cLBP orange triangles; CRPS-I grey hexagons). A. Changes in rsFC between hippocampus and precuneus/PCC are associated with changes in pain intensity in both groups. B. Pre-treatment rsFC between NAc and postcentral gyrus is associated with changes in pain intensity in both groups.

Pre- to post-EXP changes. We also explored more intermediate changes in the rsFC between hippocampus and precuneus/PCC by correlating the changes in pain from pre- to post-EXP with changes in hippocampus-precuneus/PCC rsFC from pre- to post-EXP. There was only a weak correlation (r = .30, p = .24).

Associations between pre-treatment data and reductions in pain intensity

Self-reported data. There were no significant correlations between reductions in pain intensity and age (r = -.29, p = .20), pain duration (r = -.10, p = .67), and there were no significant differences between the males and females ($F_{1,18}$ = 1.53, p = .23), pain types ($F_{1,18}$ = 1.11, p = .31), and medication use at baseline ($F_{1,18}$ = 0.07, p = .79).

There were also no significant correlations between pain relief and any pre-treatment pain-related variable: pain intensity (r = .40, p = .08), pain-related fear (r = .08, p = .72), pain catastrophizing (r = .39, p = .09), pain-related disability (r = .18, p = .45).

Changes in pain intensity were also not correlated with days of treatment (r = -.09, p = .70), days between start of treatment and follow-up assessment (r = -.004, p = .99), and did not relate to medication changes from pre- to FU-EXP ($F_{1,15}$ = 0.72, p = .41).

rsFC data.

Pain type. When taking the cLBP and CRPS-I group separately, both groups showed a strong correlation between pain relief and pre-treatment rsFC between NAc and postcentral gyrus (patients with cLBP: r = .81; patients with CRPS-I: r = .86) (**Figure S2B**).

Pre- to post-EXP changes. We also explored whether pre-EXP rsFC would be associated with more intermediate changes in pain intensity (from pre- to post-EXP). Correlation analyses showed a strong, significant correlation (r = .70, p = .001).

Supplemental References

1. Harden RN, Bruehl S, Perez RS, Birklein F, Marinus J, Maihofner C, et al. (2010): Validation of proposed diagnostic criteria (the "Budapest Criteria") for Complex Regional Pain Syndrome. *Pain*. 150:268-274.
2. Arrindell WA, Ettema JHM (2003): *SCL-90. Handleiding bij een multidimensionele psychopathologie-indicator*. Lisse: Swets & Zeitlinger.
3. Whitfield-Gabrieli S, Nieto-Castanon A (2012): Conn: a functional connectivity toolbox for correlated and anticorrelated brain networks. *Brain Connect*. 2:125-141.
4. Penny WD, Friston KJ, Ashburner JT, Kiebel SJ, Nichols TE (2011): *Statistical parametric mapping: the analysis of functional brain images*. Elsevier.
5. Behzadi Y, Restom K, Liau J, Liu TT (2007): A component based noise correction method (CompCor) for BOLD and perfusion based fMRI. *Neuroimage*. 37:90-101.
6. Hayes AF (2017): *Introduction to mediation, moderation, and conditional process analysis: A regression-based approach*. New York: Guilford Press.
